# Synthesis, Characterization, and Toxicity Evaluation of Size-Dependent Iron-Based Metal–Organic Frameworks

**DOI:** 10.3390/nano15120927

**Published:** 2025-06-14

**Authors:** Zhang Liu, Huaiyu Deng, Yuanzhi Zheng, Yuan Tian, Yanting Zhang, Renz Marion Garcia, Sheena Anne Henson Garcia, King Lun Yeung

**Affiliations:** 1 Division of Environment and Sustainability, The Hong Kong University of Science and Technology, Clear Water Bay, Kowloon, Hong Kong, China; kezliu@ust.hk (Z.L.);; 2 HKUST Shenzhen Research Institute, Hi-Tech Park, Shenzhen 518057, China; 3 Department of Chemical and Biological Engineering, The Hong Kong University of Science and Technology, Clear Water Bay, Kowloon, Hong Kong, China

**Keywords:** MOFs toxicity, size dependent, cell viability, MTT, ROS

## Abstract

Iron-based metal–organic frameworks (Fe-MOFs) are promising for biomedical and environmental applications due to their porosity, tunable chemistry, and biocompatibility. This study examines how particle size, morphology, and ligand composition affect the properties and cytotoxicity of MIL-101(Fe) and MIL-88A. MIL-101(Fe) (octahedral) and MIL-88A (rod-like) were synthesized with a controlled size (~200 nm to ~5 μm). Both showed a high crystallinity and stability. Cytotoxicity assays in A549 cells revealed size- and structure-dependent effects: smaller particles of MIL-88A caused greater toxicity (32.5% viability) than MIL-101(Fe) (66.1% viability at 100 μg/mL), while larger particles were less toxic. MIL-88A also induced higher reactive oxidative species (ROS) levels and degraded more rapidly, releasing more Fe ions. Toxicity predication analysis indicated the higher inherent toxicity of MIL-88A’s ligand (fumaric acid) compared to MIL-101(Fe)’s terephthalic acid. These results demonstrate that structural and chemical factors collectively influence Fe-MOFs’ biocompatibility and highlight the importance of rational design for safer MOF applications.

## 1. Introduction

Metal–organic frameworks (MOFs) are a diverse class of crystalline porous materials constructed from metal nodes coordinated to organic linkers, offering exceptional structural tunability and surface functionality [1,2]. Since their introduction by Yaghi et al. in the late 1990s [3], MOFs have attracted increasing attention for applications ranging from gas storage and separation to catalysis, sensing, and drug delivery [4,5]. Among their defining characteristics are ultra-high surface areas (often exceeding 2000 m^2^/g), adjustable pore sizes, and the ability to incorporate various functional groups, making them especially promising for biomedical applications [6]. In particular, nanoscale MOFs (NMOFs) have emerged as powerful candidates in drug delivery, imaging, and theranostics [7], owing to their enhanced cellular uptake, controlled biodegradability, and efficient loading of therapeutic agents.

Iron-based MOFs (Fe-MOFs), such as MIL-100(Fe), MIL-101(Fe), and MIL-88A(Fe), are representative ones due to their relative biocompatibility, low cost, and favorable degradation profiles under physiological conditions [8]. Compared to MOFs based on zinc or zirconium, Fe-MOFs generally exhibit a lower intrinsic cytotoxicity and are often considered safer for biomedical use. For instance, both in vitro and in vivo studies have reported relatively low adverse effects for MIL-100(Fe) and MIL-88A(Fe) at relatively high concentrations [9,10,11], suggesting that the nature of the metal center and linker chemistry critically governs their biological interactions. Despite this, emerging evidence indicates that MOFs’ cytotoxicity is not uniform across all compositions and morphologies [12,13,14]. Factors such as particle size, shape, surface area, ligand identity, and degradation kinetics significantly influence cellular responses. Notably, smaller MOF particles show a higher cellular internalization and often elicit stronger toxic effects, partly due to the generation of reactive oxygen species (ROS) via Fenton-like reactions induced by MOFs [15,16]. MIL-101(Fe), built from 1,4-benzenedicarboxylate (BDC) linkers, has demonstrated selective toxicity in cancer cells such as HeLa and A549 [17,18,19], whereas analogues like MIL-88A (with fumarate linkers) generally show a low cytotoxicity under comparable conditions [14,18,20]. The influence of ligand chemistry is particularly striking: dicarboxylate aromatic ring-based ligands and carbon chain-based ligands can significantly attenuate MOFs’ cytotoxic effects, pointing to the role of stability, surface reactivity, and degradation behavior in modulating cell compatibility. Furthermore, the structural flexibility and morphology of MOFs also impact cellular uptake and immune clearance. For example, the capsule-like, deformable nature of MIL-88A(Fe) may reduce recognition by phagocytes and minimize inflammatory responses [21].

In this study, we investigate the size- and structure-dependent cytotoxicity of two Fe-based MOFs, MIL-101(Fe) and MIL-88A(Fe), synthesized with controlled variations in particle size and morphology. MIL-101(Fe) was prepared in three size regimes (~1 μm, 500 nm, and 200 nm), while MIL-88A was synthesized in ~3 μm, 5 μm, and 10 μm forms. Using the A549 human lung epithelial cell line as a model, the cell viability via MTT assay and oxidative stress via intracellular ROS measurements were assessed. These assays provide insights into the cytocompatibility of different Fe-MOF architectures and elucidate how physicochemical parameters influence biological outcomes. By systematically correlating particle size and framework structure with toxicity profiles, this work contributes to a deeper understanding of Fe-MOF nanotoxicology and informs the design of safer, application-specific MOFs for biomedical use.

## 2. Materials and Methods

### 2.1. Chemicals

For material synthesis: Iron(III) chloride hexahydrate (FeCl_3_·6H_2_O, 99%, Sigma-Aldrich, St. Louis, MO, USA), terephthalic acid (98%, Sigma-Aldrich), fumaric acid (≥99%, Sigma-Aldrich, USA), N,N-dimethylformamide (DMF, anhydrous, 99.8%, Sigma-Aldrich, methanol (anhydrous, 99.8%, Sigma-Aldrich, ethanol (ACS reagent, ≥99.5%, Sigma-Aldrich, and deionized water (resistivity 18.2 MΩ·cm, Millipore system, DE) for the synthesis of Fe-MIL-101 and Fe-MIL-88A.

For cytotoxicity evaluation: Human lung epithelial A549 cells (CCL-185™, ATCC, Manassas, VA, USA) were cultured in Dulbecco’s Modified Eagle Medium/Nutrient Mixture F-12 (DMEM/F12, high glucose; Gibco, Waltham, MA, USA) supplemented with 10% heat-inactivated, certified fetal bovine serum (FBS; Gibco) and 1% penicillin–streptomycin (100×; Gibco). Dulbecco’s phosphate-buffered saline (DPBS, without calcium and magnesium, pH 7.4; Gibco) was used for washing procedures. Cell viability was determined using the MTT assay with 3-(4,5-dimethylthiazol-2-yl)-2,5-diphenyltetrazolium bromide (MTT, ≥98%; Sigma-Aldrich) and dimethyl sulfoxide (DMSO, ≥99.9%; Sigma-Aldrich). Intracellular reactive oxygen species (ROS) levels were quantified using 2′,7′-dichlorodihydrofluorescein diacetate (DCFH-DA, ≥97%; Sigma-Aldrich). All reagents were used as received without further purification.

### 2.2. Synthesis of MOFs

#### 2.2.1. Synthesis of MIL-101(Fe) Nanoparticles

MIL-101(Fe) nanoparticles were synthesized via a solvothermal method. In a typical synthesis, 1.35 g of FeCl_3_·6H_2_O and terephthalic acid were dissolved in 60 mL of N,N-dimethylformamide (DMF) under stirring. The mixture was then transferred to a Teflon-lined stainless steel autoclave (SH, China) and heated at 150 °C for 8 h. Upon cooling to room temperature, the resulting precipitate was collected by centrifugation, washed repeatedly with DMF and methanol to remove unreacted species and residual solvent, and dried at 80 °C for 12 h. To investigate the effect of ligand concentration on particle size, three samples were prepared using increasing amounts of terephthalic acid, 0.412 g, 1.236 g, and 1.648 g, while keeping all other parameters constant (1.35 g FeCl_3_·6H_2_O, 60 mL DMF, 150 °C, 8 h), resulting in metal/ligand ratios of 2:1, 2:3, and 2:4, respectively. These samples are hereafter referred to as MIL-101(Fe)(M/L = 2:1), MIL-101(Fe)(M/L = 2:3), and MIL-101(Fe)(M/L = 2:4), respectively.

#### 2.2.2. Synthesis of Fe-MIL-88A Nanoparticles

Fe-MIL-88A nanoparticles were synthesized via a hydrothermal route. Briefly, 2.2722 g (8.4 mmol) of FeCl_3_·6H_2_O and 0.9744 g (8.4 mmol) of fumaric acid was dissolved in 42 mL of deionized water and stirred for 1 h to ensure complete dissolution. The resulting clear solution was sealed in a media bottle and heated in a conventional oven at 105 °C for 12 h. After the reaction, the solid product was collected by centrifugation, thoroughly washed with deionized water followed by ethanol, and dried under reduced pressure at 100 °C. To examine the influence of reaction temperature on particles’ morphology and size, two additional samples were synthesized using identical chemical compositions but reaction temperatures of 85 °C and 65 °C, respectively. These samples are designated as MIL-88A (105 °C), MIL-88A (85 °C), and MIL-88A (65 °C).

### 2.3. Characterizations

The synthesized Fe-MIL-101 and Fe-MIL-88A nanoparticles were characterized to confirm their crystal structure, morphology, particle size, and thermal stability. Powder X-ray diffraction (XRD) patterns were collected using a Bruker D8 diffractometer (Billerica, MA, USA) with Cu Kα1 radiation (2θ = 4–45°) to verify the crystalline phases and purity of the MOFs. Scanning electron microscopy (SEM, JEOL JSM-7100F, Tokyo, Japan) was employed to observe the particles’ morphology and size distribution; samples were ethanol-dispersed, drop-cast on silica wafers, gold-coated, and imaged at 10 kV. Dynamic light scattering (DLS) was used to assess the hydrodynamic diameters and dispersion behavior of the MOF nanoparticles in aqueous media. Thermogravimetric analysis (TGA, PerkinElmer, Waltham, MA, USA) was conducted in air from room temperature to 800 °C at 10 °C/min to evaluate thermal stability and framework integrity. Transmission electron microscopy (TEM, JEM-2010F, NY, USA) further confirmed the particles’ morphology at high resolution using vacuum-dried suspensions on carbon-coated copper grids. These analyses collectively confirmed the successful synthesis and structural integrity of Fe-MIL-101 and Fe-MIL-88A with tunable particle sizes.

### 2.4. Cytotoxicity Evaluation

#### 2.4.1. Cell Culture

Human lung adenocarcinoma A549 cells were cultured in standard growth conditions (37 °C, 5% CO_2_, 90% humidity) in an appropriate culture medium. The cells were seeded in 96-well plates at a density of 5 × 10^4^ to 1 × 10^5^ cells/mL and incubated for 12–18 h to allow for attachment prior to nanoparticle treatment.

#### 2.4.2. MTT Assay

Cell viability following exposure to Fe-MIL-101 and Fe-MIL-88A nanoparticles was evaluated using the MTT assay. A549 cells were treated with a range of nanoparticle concentrations (1.56, 3.125, 6.25, 12.5, 25, 50, and 100 μg/mL) for 24 h. Subsequently, MTT solution (5 μg/mL) was added to each well and incubated for 4 h at 37 °C. The resulting formazan crystals were solubilized in 150 μL of DMSO, and absorbance was measured at 490 nm using a microplate reader (SpectraMax 190; Molecular Devices). All experiments were performed in triplicate, and cell viability was expressed as a percentage relative to untreated controls.

#### 2.4.3. Reactive Oxygen Species Assay

Reactive oxygen species (ROS) generation was quantified using 2′,7′-dichlorodihydrofluorescein diacetate (DCFH-DA). A549 cells were exposed to Fe-MIL-101 and Fe-MIL-88A nanoparticles at concentrations of 6.25, 12.5, and 25 μg/mL for 24 h. Following treatment, the culture medium was removed, and the cells were washed with 200 μL of fresh DMEM/F12. DCFH-DA solution (100 μL, 40 μM) was then added to each well, and the cells were incubated for 30 min at 37 °C. After incubation, the cells were washed twice with 400 μL of DPBS to remove excess dye, and 200 μL of DPBS was added to each well. Fluorescence intensity was measured at excitation/emission wavelengths of 485/527 nm using a microplate reader. ROS levels were normalized to untreated controls and expressed as relative fluorescence intensity.

## 3. Results and Discussion

### 3.1. Synthesis of Size-Dependent Fe-MOFs

**Scheme 1:**
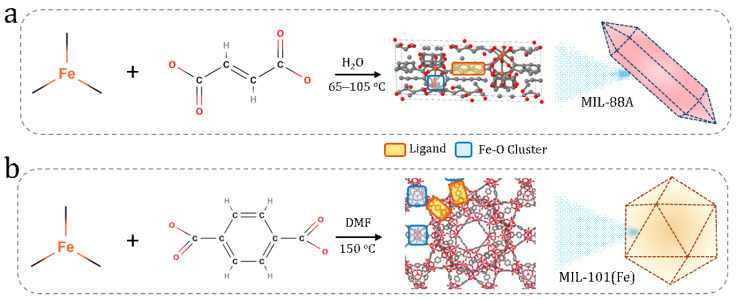
Schematic illustration of the synthesis routes for MIL-88A (**a**) and MIL-101(Fe) (**b**).

MIL-88A and MIL-101(Fe), two structurally distinct iron-based MOFs, were synthesized via solvothermal methods using the same iron(III) precursor but varied organic ligands of fumaric acid (FA) or terephthalic acid (H_2_BDC), respectively. As shown in Scheme 1, MIL-88A was obtained by reacting FeCl_3_·6H_2_O with FA in deionized water at temperatures ranging from 65 °C to 105 °C, yielding rod-shaped crystals whose dimensions decreased with increasing synthesis temperatures. MIL-101(Fe) was synthesized in DMF at 150 °C, with size control (1–5 μm) achieved by adjusting the Fe:H_2_BDC molar ratios (i.e., 2:1, 2:3, and 2:4), which produced progressively smaller particles (200 nm–1 μm). These methods enabled precise control over crystal morphology and particle size without compromising the framework’s integrity. Structurally, MIL-88A features a flexible breathing framework responsive to environmental stimuli [22,23], while MIL-101(Fe) exhibits a rigid architecture with an exceptionally high surface area and pore volume [19,24]. Both MOFs display a moderate aqueous stability, with degradation products of iron ions and dicarboxylate ligands that are considered to have a relatively low toxicity at low concentrations. However, their interactions with biological systems can vary significantly depending on properties [25] such as particle size, dimension, and metal release kinetics, all of which can influence cellular oxidative stress, viability, and membrane integrity. Therefore, these two Fe-MOFs were selected as model materials to investigate structure-dependent toxicity in cellular environments, with the goal of informing the safe design and uses of MOFs for environmental and biomedical applications.

### 3.2. Characterizations of Fe-MOF Nanoparticles

**Figure 1:**
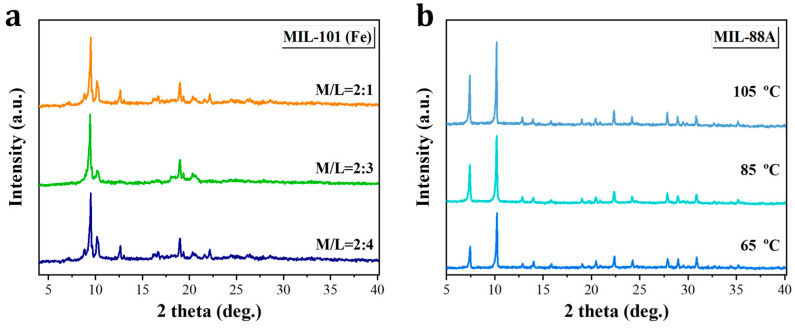
The XRD patterns of MIL-101(Fe) (**a**) and MIL-88A (**b**) synthesized at varied temperatures or at varied M/L ratios.

The XRD patterns of both MIL-88A and MIL-101(Fe) shown in Figure 1 exhibit sharp and well-defined peaks, indicative of their high crystallinity. For MIL-101(Fe), increasing the concentration of the organic ligand (H_2_BDC) led to a slight reduction in peak intensity and peak broadening, particularly at an M/L ratio of 2:4. This suggests the formation of smaller crystalline domains under ligand-rich conditions. Similarly, elevating the synthesis temperature for MIL-88A resulted in a decreased peak intensity, indicating a reduction in crystallite size and overall crystallinity. Nevertheless, the overall diffraction features of both MIL-101(Fe) and MIL-88A remain consistent with previously reported data in the literature [17,22,26], and their main characteristic peaks can be well indexed. Importantly, no secondary phases or impurity peaks were detected in any of the samples, confirming the phase purity of the synthesized MOFs.

**Figure 2:**
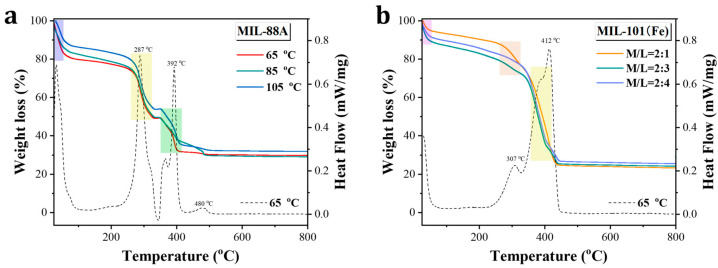
The TGA-DSC curves of MIL-88A (**a**) and MIL-101(Fe) (**b**) synthesized under varying conditions. Note: the color bocks indicate the exothermic peaks and the corresponding weigh loss ranges.

Figure 2 presents the thermogravimetric (TGA) and differential scanning calorimetry (DSC) analyses of MIL-88A and MIL-101(Fe), showing their thermal stability and decomposition behavior. For MIL-88A (Figure 2a), three major weight loss stages are observed. The initial mass loss (~20.4 wt%) below 80 °C corresponds to the evaporation of adsorbed water and residual solvents. A second stage at ~287 °C (~30 wt%) is attributed to the dissociation of coordination bonds between Fe(III) centers and carboxylate groups from fumaric acid, indicating structural destabilization. The final stage around 392 °C (~20 wt%) corresponds to the thermal decomposition of the organic ligand, marking complete framework collapse. The DSC curve further supports these transitions, showing exothermic peaks that align with solvent release and framework decomposition events.

On the other hand, MIL-101(Fe) synthesized at an Fe:H_2_BDC ratio of 2:1 (Figure 2b) also displays three distinct thermal events. An initial minor weight loss below 80 °C indicates the removal of solvent and moisture. A second mass loss (~12 wt%) around 307 °C corresponds to the breaking of Fe–BDC coordination bonds, while a substantial third loss (~30 wt%) near 312 °C is associated with the pyrolysis of the terephthalic acid linker. The corresponding DSC data reflect similar thermal transitions, validating the stepwise degradation behavior. Overall, both MOFs exhibit a good thermal stability up to ~300 °C, with MIL-88A showing a slightly earlier onset of structural breakdown, likely due to differences in ligand structure and framework rigidity [24,27].

**Figure 3:**
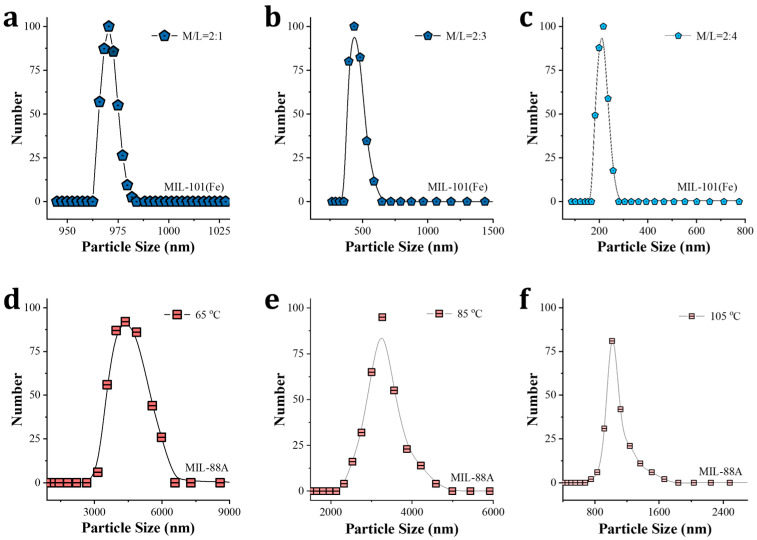
DLS curves of MIL-101(Fe) synthesized at a M/L = 2:1 (**a**), M/L = 2:3 (**b**), and M/L = 2:4 (**c**), and MIL-88A synthesized at temperatures of 65 °C (**d**), 85 °C (**e**), and 105 °C (**f**).

Dynamic light scattering (DLS) measurements were conducted at room temperature to evaluate the hydrodynamic particle size distributions of MIL-88A and MIL-101(Fe) in aqueous dispersion. Both MOF samples were dispersed in deionized water at a concentration of 0.2 mg/mL, followed by brief sonication to ensure a homogeneous suspension. The results, presented in Figure 3, demonstrate a strong influence of synthesis parameters on particle size and distribution.

For MIL-101(Fe) (Figure 3a–c), decreasing the metal-to-ligand molar ratio (Fe:H_2_BDC) from 2:1 to 2:4 led to a systematic reduction in hydrodynamic size from 948 nm to 236 nm. Specifically, the measured average sizes were 968 ± 21 nm (2:1) (Figure 3a), 487 ± 114 nm (2:3) (Figure 3b), and 236 ± 48 nm (2:4) (Figure 3c), corresponding approximately to 1 µm, 500 nm, and 200 nm, respectively. The narrow and symmetric size distributions indicate well-dispersed and relatively monodisperse particles, suggesting that a higher ligand content effectively limits particle growth; this is consistent with the classic nucleation theory that high concentrations of reactant result in fast nucleation and slow growth [28,29,30], producing relatively small crystal sizes. MIL-88A (Figure 3d–f) exhibited broader size distributions, with average hydrodynamic diameters of 4894 ± 1437 nm, 3474 ± 1062 nm, and 1128 ± 255 nm for samples synthesized at 65 °C (Figure 3d), 85 °C (Figure 3e), and 105 °C (Figure 3f), respectively, corresponding approximately to 5 µm, 3 µm, and 1 µm. The trend clearly shows that increasing the reaction temperature reduces the particle size, likely due to enhanced nucleation rates and suppressed crystal growth at higher thermal energy [31]. However, the broader size distributions suggest a less uniform morphology and higher aggregation tendencies compared to MIL-101(Fe), consistent with its rod-like structure observed in SEM analysis.

These findings confirm that the particle size and dispersion behavior of Fe-based MOFs are highly tunable through controlled synthesis parameters, which are ligand concentration for MIL-101(Fe) and reaction temperature for MIL-88A. These size variations are expected to play a critical role in determining their biological interactions and toxicity profiles, as further discussed in subsequent sections.

**Figure 4:**
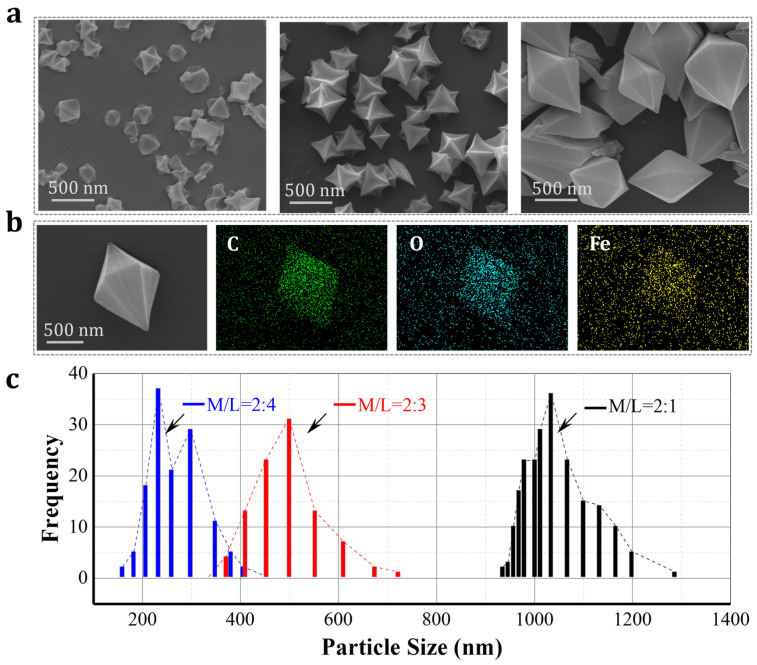
(**a**) SEM images of MIL-101(Fe) synthesized with M/L = 2:4 (**left**), 2:3 (**middle**), and 2:1 (**right**); (**b**) EDS elemental mapping images for one single particle (M/L = 2:1); and (**c**) particle size distribution measured by Image J v1.43 software (**c**).

The SEM analysis (Figure 4a) confirms the successful formation of MIL-101(Fe) with a well-defined octahedral morphology across all synthesis conditions. With increasing metal-to-ligand ratios (M/L = 2:1, 2:3, 2:4), the particle sizes reduced significantly, consistent with the DLS findings. EDS elemental mapping of MIL-101(Fe) (M/L = 2:1) shows a uniform distribution of iron, carbon, and oxygen elements throughout the particle (Figure 4b), verifying its homogeneity and suggesting a successful coordination of Fe(III) ions with terephthalic acid ligands. The ImageJ-based (software of ImageJ 1.43c) statistical analysis (Figure 4c) reveals average particle sizes of 1023 ± 86 nm, 494 ± 105 nm, and 218 ± 94 nm for M/L = 2:1, 2:3, and 2:4, respectively. These results affirm a precise size control via M/L adjustment, supporting the rationale for evaluating size-dependent toxicological responses.

**Figure 5:**
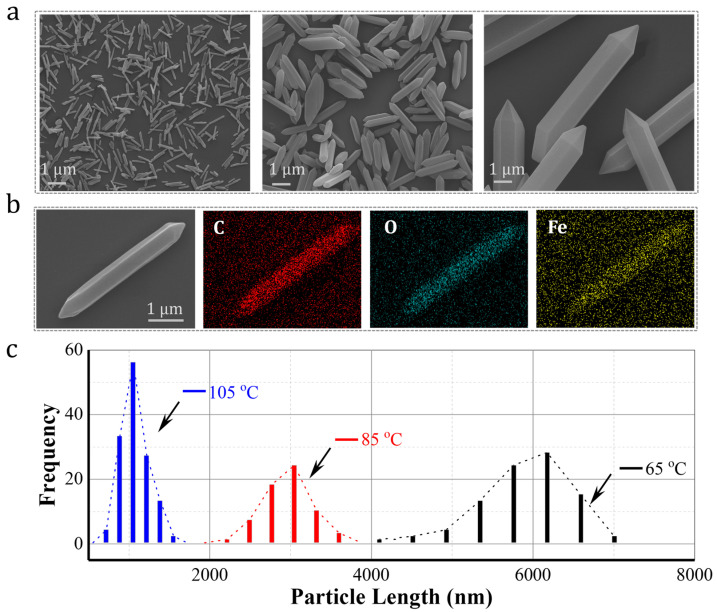
(**a**) SEM images of MIL-88A(Fe) synthesized with M/L = 2:1 (**left**), 2:3 (**middle**), and 2:4 (**right**); (**b**) EDS elemental mapping images for one single particle (M/L = 2:4); and (**c**) particle size distribution measured by Image J (v1.43) software (**c**).

MIL-88A particles (Figure 5a) exhibit characteristic rod-like morphologies, with lengths tunable by varying the synthesis temperature. SEM analysis shows that increasing the temperature from 65 °C to 105 °C results in shorter rod lengths and narrower size distributions. EDS mapping (Figure 5b) for MIL-88A synthesized at 85 °C confirms a homogeneous elemental distribution of Fe, C, and O. The ImageJ analysis (Figure 5c) indicates mean particle lengths of 5216 ± 1328 nm (65 °C), 3029 ± 705 nm (85 °C), and 1254 ± 302 nm (105 °C). These findings correlate well with the DLS measurements and support the conclusion that thermal control is an effective parameter for tuning rod length in MIL-88A. This dimensional variation is important for interpreting the materials’ cellular interactions and cytotoxicity.

### 3.3. Toxicity Evalution

**Figure 6:**
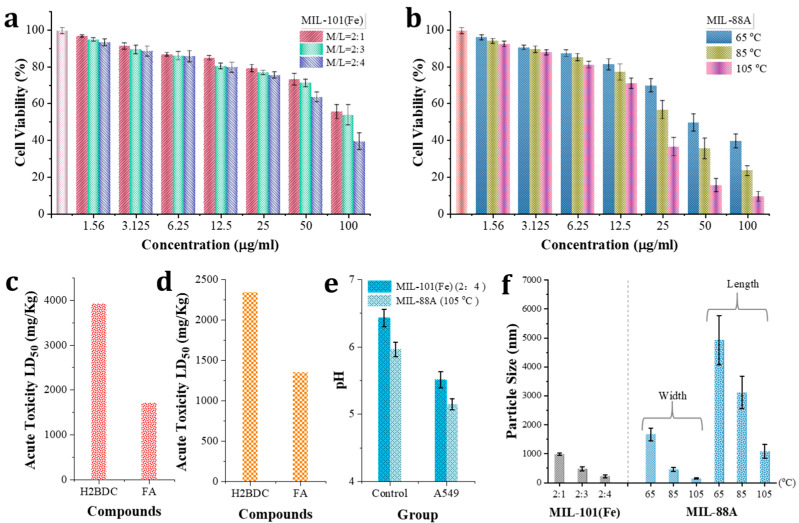
Cytotoxicity and toxicity prediction of Fe-MOFs and their precursors. (**a**) Viability of A549 cells after 24 h exposure to MIL-101(Fe) at increasing concentrations (1.56–25 μg/mL), measured via MTT assay. (**b**) Viability of A549 cells after 24 h exposure to MIL-88A under the same concentration range. (**c**) Predicted acute oral LD_50_ values (rat model) for the organic linkers terephthalic acid (H_2_BDC) and fumaric acid (FA), obtained using the Toxicity Estimation Software Tool (T.E.S.T., v5.2.1). (**d**) Predicted acute toxicity categories of H_2_BDC and FA from the ProTox-II platform. (**e**) Quantification of intracellular ROS levels in A549 cells following 24 h exposure to MIL-101(Fe) and MIL-88A at 6.25, 12.5, 25, 50, and 100 μg/mL, using the DCFH-DA assay. Fluorescence intensities were normalized to untreated controls. (**f**) Comparative ROS levels for H_2_BDC and FA at corresponding concentrations, indicating an oxidative stress response. Data represent mean ± s.d. from three independent experiments.

The MTT assay is a standard method for assessing cell viability and cytotoxicity, relying on the reduction of the yellow tetrazolium salt MTT to insoluble purple formazan crystals by mitochondrial dehydrogenases in metabolically active cells [32]. In this study, we used the MTT assay to evaluate the cytotoxic effects of MIL-101(Fe) and MIL-88A on A549 human lung epithelial cells (Figure 6a,b). Both MOFs induced dose-dependent reductions in cell viability. For MIL-101(Fe), only mild cytotoxicity was observed at lower concentrations (1.56 µg/mL: 2.6–3.4% inhibition), whereas exposure at 50 µg/mL reduced viability to 71.2–68.3%, and further to 54.3–42.4% at 100 µg/mL. Notably, formulations with smaller particle sizes (e.g., M/L = 2:4) exhibited a significantly enhanced toxicity compared to their larger counterparts, indicating a size-dependent cytotoxicity consistent with increased cellular uptake and surface reactivity [33,34].

MIL-88A exhibited a steeper toxicity profile. At 25 µg/mL, cell viability declined markedly to 69.2–37.3% and further dropped to 21.6–9.2% at 100 µg/mL. The most cytotoxic sample was the smallest-sized MIL-88A (105 °C synthesis), supporting prior findings that reduced particle dimensions increase cellular internalization and biochemical disruption [35]. The more pronounced toxicity of MIL-88A, relative to MIL-101(Fe), may also be attributed to its elongated rod-like morphology and higher aspect ratio, which have been shown to amplify cellular membrane interactions and uptake [36]. Additionally, differences in ligand chemistry (fumaric acid vs. terephthalic acid) and surface accessibility may contribute to differential reactivity and degradation [37].

To further probe the molecular basis of toxicity, we examined the predicted acute oral LD_50_ values of the organic ligands used in MOF synthesis, which are terephthalic acid and fumaric acid, via the Toxicity Estimation Software Tool (T.E.S.T.) and ProTox-II (Figure 6c,d) [38]. Both platforms indicated a higher toxicity for FA (LD_50_ = 1350 or 1758 mg/kg) than for terephthalic acid (LD_50_ = 2365 or 3796 mg/kg), though both ligands fall within moderate toxicity ranges comparable to several food additives and pharmaceuticals [39,40]. While the ligands are not inherently cytotoxic at experimental concentrations, their release upon MOFs’ degradation could contribute to the observed effects, particularly for MIL-88A. On the other hand, medium acidification was also observed following MOF treatment. At 50 µg/mL, the culture pH decreased to 5.43 for MIL-101(Fe) and 5.14 for MIL-88A, compared to pH ~6.5 in untreated controls. Such acidification is likely driven by Fe^3+^ ion release and the consumption of hydroxide ions during hydrolytic degradation, leading to an environment unfavorable for cell survival and metabolic function. Intracellular acidosis has been previously linked to mitochondrial dysfunction and apoptosis in epithelial cells [41].

The geometric surface areas (SAs) of different MOF particles were further estimated to better understand their exposure profiles. MIL-101(Fe) particles were approximated as octahedra; taking the 200 nm particle (M/L = 2:4) as a unit SA baseline, the 500 nm and 1 µm particles exhibit ~1/25 and ~1/100 of that surface area, respectively. MIL-88A particles, modeled as tapered rods, demonstrated an inverse relationship: the 65 °C sample (~5 µm) has ~3.33× the SA of the smaller 105 °C sample (~1.25 µm), enhancing its potential for degradation and cellular interaction. These trends are in line with prior studies showing that a higher surface area facilitates faster ion and ligand release, elevating oxidative stress and inflammatory responses [42,43].

Taken together, these results demonstrate that the cytotoxicity of Fe-based MOFs is governed by a multifactorial interplay of particle size, surface geometry, ligand identity, and concentration. Smaller particles with higher surface-to-volume ratios and more reactive ligands, particularly MIL-88A, exhibit increased toxicity, likely due to enhanced cellular uptake, greater ion release, and local acidification. These insights are essential for guiding the safe design of MOF-based biomedical or environmental applications.

**Figure 7:**
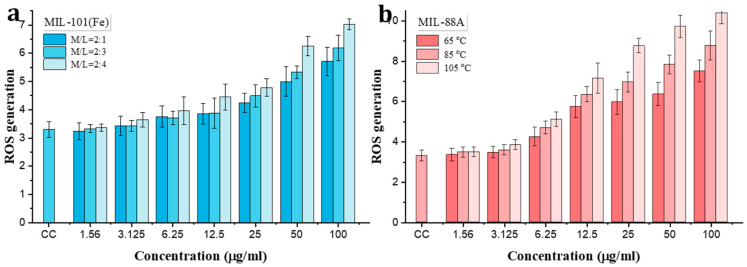
Intracellular ROS generation in A549 cells induced by MIL-101 synthesized at a M/L = 2:1, 2:3, and 2:4, and MIL-88 synthesized at temperatures of 65 °C, 80 °C, and 105 °C. (**a**) ROS levels in A549 cells after 24 h exposure to increasing concentrations of MIL-101(Fe) (1.56–25 μg/mL), measured by DCFH-DA fluorescence assay. (**b**) ROS levels in A549 cells after 24 h exposure to MIL-88A under the same concentration range. Fluorescence intensity values were normalized to untreated controls. Both MOFs induced a concentration-dependent increase in intracellular ROS, with MIL-88A eliciting a stronger oxidative stress response than MIL-101(Fe), particularly at higher concentrations and with smaller-sized particles. Data represent mean ± s.d. of three independent experiments (*n* = 3).

ROS are key indicators of oxidative stress and play a central role in nanoparticle-induced cytotoxicity. The DCFH-DA assay enables the quantification of intracellular ROS levels by detecting the oxidation of non-fluorescent DCFH to fluorescent DCF in response to ROS activity [44]. Elevated ROS can disrupt cellular homeostasis, damage macromolecules, and trigger apoptotic pathways [45,46]. In this study, we assessed the oxidative stress induced by MIL-101(Fe) and MIL-88A in A549 lung epithelial cells (Figure 7a,b), as a mechanistic contributor to the observed cytotoxicity (Figure 6).

For MIL-101(Fe), ROS levels remained near baseline at low concentrations (1.56 µg/mL, fluorescence intensity ~3.4 vs. control ~3.3) but increased progressively with dosage. At 25 µg/mL, ROS levels reached ~4.8 and continued to rise to ~6.3 at 50 µg/mL and ~6.9 at 100 µg/mL. This trend was more pronounced in samples with smaller particle sizes (e.g., M/L = 2:4), suggesting a correlation between surface area and ROS production, likely due to accelerated degradation kinetics and the greater exposure of Fe coordination sites [47]. In contrast, MIL-88A induced markedly higher ROS levels across all tested concentrations. Even at 1.56–3.125 µg/mL, fluorescence intensities ranged from ~3.4 to ~3.7. A sharp escalation occurred beyond 6.25 µg/mL, with ROS reaching ~5.1 at 6.25 µg/mL and peaking at ~10.3 at 100 µg/mL for the smallest particles (~1 µm). Compared to MIL-101(Fe), MIL-88A generated significantly more ROS at equivalent concentrations and sizes, highlighting its stronger oxidative stress potential. The observed oxidative stress can be attributed in part to iron ion release from both Fe-based MOFs under physiological and mildly acidic conditions. Fe^2+^/Fe^3+^ ions are known to catalyze Fenton reactions, in which hydrogen peroxide (H_2_O_2_) is converted into highly reactive hydroxyl radicals (•OH), amplifying oxidative damage [48]. Indeed, certain Fe-MOFs such as NH2-MIL-88B have been reported to exhibit intrinsic peroxidase-like activity, facilitating rapid •OH generation via Fenton chemistry [49,50]. This suggests that both MIL-101(Fe) and MIL-88A could act as nanozymes under intracellular conditions, accelerating ROS’ formation and redox imbalance.

In addition to catalytic activity, elevated intracellular iron concentrations may overwhelm homeostatic mechanisms, impairing iron-regulated enzymes, altering redox-sensitive signaling pathways, and contributing to ferroptosis [51,52]. MIL-88A, which is more prone to structural degradation and Fe release due to its rod-like morphology and lower thermal stability [43], appears particularly potent in this regard. In addition, ligand chemistry may further modulate toxicity. Terephthalic acid, used in MIL-101(Fe), has been reported to possess selective cytotoxicity against tumor cells, potentially enhancing therapeutic utility but also contributing to basal toxicity. Fumaric acid, the organic linker in MIL-88A, is a naturally occurring metabolite with known immunomodulatory properties [53], which may reduce immunogenicity but does not preclude oxidative effects when released in excess. These findings suggest that the oxidative stress induced by Fe-MOFs is influenced by a combination of factors: particle size, morphology, Fe ion release, catalytic ROS generation, and ligand composition. MIL-88A, in particular, poses a greater oxidative risk due to its higher reactivity and structural instability. These insights underscore the importance of tailoring MOFs’ physicochemical properties to minimize cellular damage while leveraging therapeutic functionalities.

**Figure 8:**
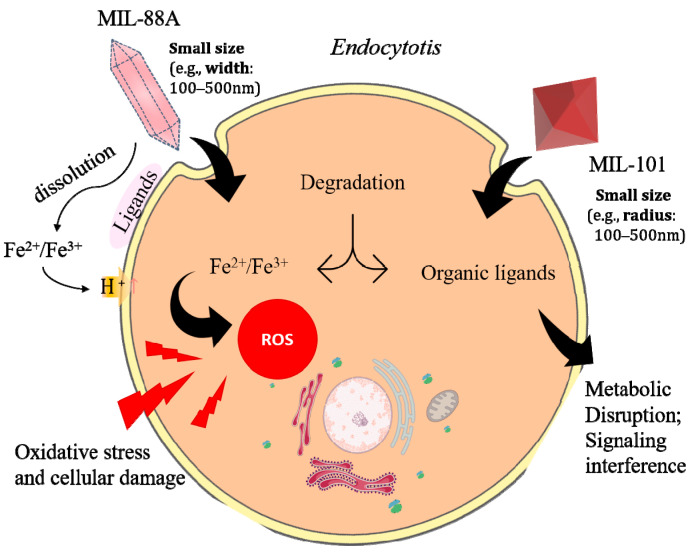
Schematic illustration of probable cell toxicity mechanisms induced by varied MOFs. MIL-101(Fe) and MIL-88A nanoparticles are internalized by cells via endocytosis. Upon intracellular degradation, both MOFs release Fe^2+^/Fe^3+^ ions and organic ligands (e.g., terephthalic acid or fumaric acid). The released iron catalyzes Fenton-like reactions, generating ROS, which lead to oxidative stress and cellular damage. Ligand fragments may further contribute to toxicity via metabolic disruption or signaling interference. Compared to MIL-101(Fe), MIL-88A has a smaller size and rod-shaped morphology and exhibits enhanced degradation and ROS generation, contributing to its greater cytotoxic potential.

Based on the experimental findings, the cytotoxicity of Fe-based MOFs, particularly MIL-101(Fe) and MIL-88A, arises from a multifactorial interplay involving particle size, morphology, chemical composition, and intracellular degradation dynamics. Both cell viability (MTT assay) and oxidative stress (ROS assay) measurements reveal distinct patterns that shed light on the underlying toxicity mechanisms. As illustrated in the schematic model (Figure 8), these effects align with established principles in nanoparticle toxicology and redox biology. On the basis of prior studies, it is known that smaller particles (e.g., ~200 nm MIL-101(Fe)) exhibited greater cytotoxicity than their larger counterparts in MTT assays, likely due to enhanced cellular uptake via clathrin- or caveolae-mediated endocytosis [54], as was also evidenced by other research [55]. Upon internalization, these particles may accumulate in lysosomes and mitochondria, where an acidic pH and enzymatic activity promote partial degradation, releasing Fe^3+^ and organic ligands. This can lead to impaired mitochondrial membrane potential, ATP depletion, or lysosomal rupture; these events contribute to metabolic dysfunction and cell death [56]. Notably, this enhanced cytotoxicity was not always paralleled by elevated ROS levels, indicating that non-ROS-dependent mechanisms, such as mechanical stress, organelle disruption, or autophagy dysregulation, may also play dominant roles in the toxicity of smaller Fe-MOFs. Conversely, larger Fe-MOF particles, specifically MIL-88A with its elongated rod-like geometry, triggered a robust ROS generation without a proportional reduction in cell viability. This suggests that oxidative stress in these cases may arise from extracellular or membrane-associated pathways. Due to size constraints, large particles are less readily internalized, yet they can interact extensively with the cell membrane. The pointed, anisotropic structure of MIL-88A may induce localized membrane deformation or activate surface-bound redox enzymes such as NADPH oxidase, promoting lipid peroxidation and redox signaling [47,57]. Additionally, the extracellular degradation of MIL-88A in slightly acidic microenvironments (pH ~6.0~6.5) releases Fe^3+^ ions capable of catalyzing Fenton-like reactions with ambient H_2_O_2_, generating highly reactive hydroxyl radicals (•OH) that diffuse into the cell and amplify oxidative stress [48].

The spatial location of ROS generation, whether intracellular, lysosomal, or extracellular, thus plays a pivotal role in shaping toxicity profiles. While intracellular ROS can initiate apoptosis via mitochondrial pathways, extracellular ROS can impair membrane integrity or activate pro-inflammatory signaling cascades. The accessibility and catalytic activity of redox-active iron species determine the extent and nature of oxidative stress induced by MOF degradation. Furthermore, ligand composition significantly influences toxicity [12]. MIL-88A contains fumaric acid, which is more hydrophilic and potentially more toxic than terephthalic acid in MIL-101(Fe). This, combined with the higher degradability of MIL-88A, leads to greater ligand and ion release, contributing to both cellular stress and acidification of the medium (pH ~5.14). Taken together, these findings support a nuanced toxicity model where Fe-MOF-induced cytotoxicity is governed by (1) endocytosis-mediated internalization and subcellular damage in smaller particles; (2) surface interaction and extracellular ROS production in larger or rod-shaped particles; and (3) ligand identity and release kinetics contributing to both ROS-dependent and independent pathways. Therefore, a comprehensive evaluation of Fe-MOFs’ safety must consider not only composition and size but also shape, degradation kinetics, and cellular interaction dynamics.

## 4. Conclusions

This study demonstrates that the physicochemical properties of Fe-based MOFs—including particle size, morphology, and ligand composition—critically influence their cytotoxicity toward A549 cells. MIL-101(Fe) with an octahedral morphology and MIL-88A with a rod-like shape were synthesized in tunable sizes ranging from ~200 nm to ~5 μm by adjusting reaction parameters. Cytotoxicity assays revealed that nanoscale particles (e.g., MIL-101(Fe) and MIL-88A) exhibited significantly greater toxicity than their larger counterparts; this trend was consistent across concentrations, highlighting the critical role of particle size and shape. In addition, other factors such as composition also greatly affect toxicity; for example, a large sized MIL-88A reduces cell viability to 32.5% at 100 μg/mL, compared to 66.1% for MIL-101(Fe) at the same dose. Intracellular ROS levels, measured via DCFH-DA fluorescence, showed a 1.72-fold increase in the MIL-88A (85 °C) group relative to the control, suggesting oxidative stress as a key mechanism of toxicity. pH measurements of degradation media further confirmed the faster acidification and degradation of MIL-88A, implying a higher Fe ion release, which may compound its toxicity. Computational predictions also indicated that fumaric acid, the ligand in MIL-88A, possesses a higher inherent toxicity than the terephthalic acid used in MIL-101(Fe). Taken together, these results indicate that both structural characteristics and chemical composition govern the biological effects of Fe-MOFs, with the smaller, rod-shaped MIL-88A exhibiting the most pronounced toxicity due to enhanced cellular uptake, faster degradation, and ligand-specific effects. These insights are critical for guiding the rational design of safer MOFs for biomedical and environmental applications.

## Data Availability

The original contributions presented in this study are included in the article. Further inquiries can be directed to the corresponding authors (kekyeung@ust.hk).
